# Symptomatology, prognosis, and clinical findings of Monkeypox infected patients during COVID‐19 era: A systematic‐review

**DOI:** 10.1002/iid3.722

**Published:** 2022-10-11

**Authors:** Vikash Jaiswal, Priyanshu Nain, Dattatreya Mukherjee, Amey Joshi, Mittal Savaliya, Angela Ishak, Nitya Batra, Dipansha Maroo, Deepak Verma

**Affiliations:** ^1^ Department of Research Larkin Community Hospital South Miami Florida USA; ^2^ Department of Medicine Maulana Azad Medical College New Delhi India; ^3^ Department of Medicine Raiganj Government Medical College and Hospital Raiganj West Bengal India; ^4^ Department of Medicine GMERS Medical College Junagadh Gujarat India; ^5^ Department of Research Larkin Community Hospital South Miami Fl USA; ^6^ Department of Medicine Janaki Medical College Dhanusha Nepal

**Keywords:** infection, monkeypox virus, outcomes, pathology

## Abstract

**Background:**

The recent outbreak of Human Monkeypox (MPXV) in nonendemic regions of the world is of great concern.

**Objective:**

We aimed to systematically analyze the current epidemiology, clinical presentation, and outcomes of the Monkeypox virus.

**Method:**

Systematic literature was conducted in PubMed, Embase, Google Scholar, and Scopus using predefined MESH terms by using “AND” and “OR.” The following search terms were used: Monkeypox [MeSH] OR “Monkeypox virus” [MeSH] OR “POX” OR “Monkeypox” AND “Outbreak” AND “Outcomes” from December 2019 till 14th June 2022 without restrictions of language.

**Results:**

A total of 1074 (99.90%) patients tested positive for Monkeypox virus through RT‐PCR while 1 (0.09) patient was suspected. There was a gender difference with male predominance (54.23% vs. 45.48%) compared with female patients. Mean age (±*SD*) of patients was 20.66 ± 16.45 years. The major symptoms were rash (100%), fever (96%), and other important symptoms were upper respiratory symptoms (97%), headache (95%), vomiting (95%), oral ulcers (96%), conjunctivitis (96%) and lymphadenopathy (85%). The average mean duration of treatment was 5 days, while the mean hospitalization duration was 13.3 ± 6.37 days. The outcome of 20 patients was available, 19 of 20 patients recovered fully from monkeypox, however, 1 patient was not able to survive resulting in death.

**Conclusion:**

The recent monkeypox virus outbreak has shown that the virus could transmit in ways that were not previously expected. Further research is needed to understand the possible outcomes and association with humans and their different organ systems.

## Graphical abstract

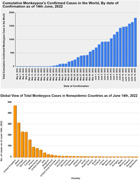

## 1 INTRODUCTION

**Figure 1:**
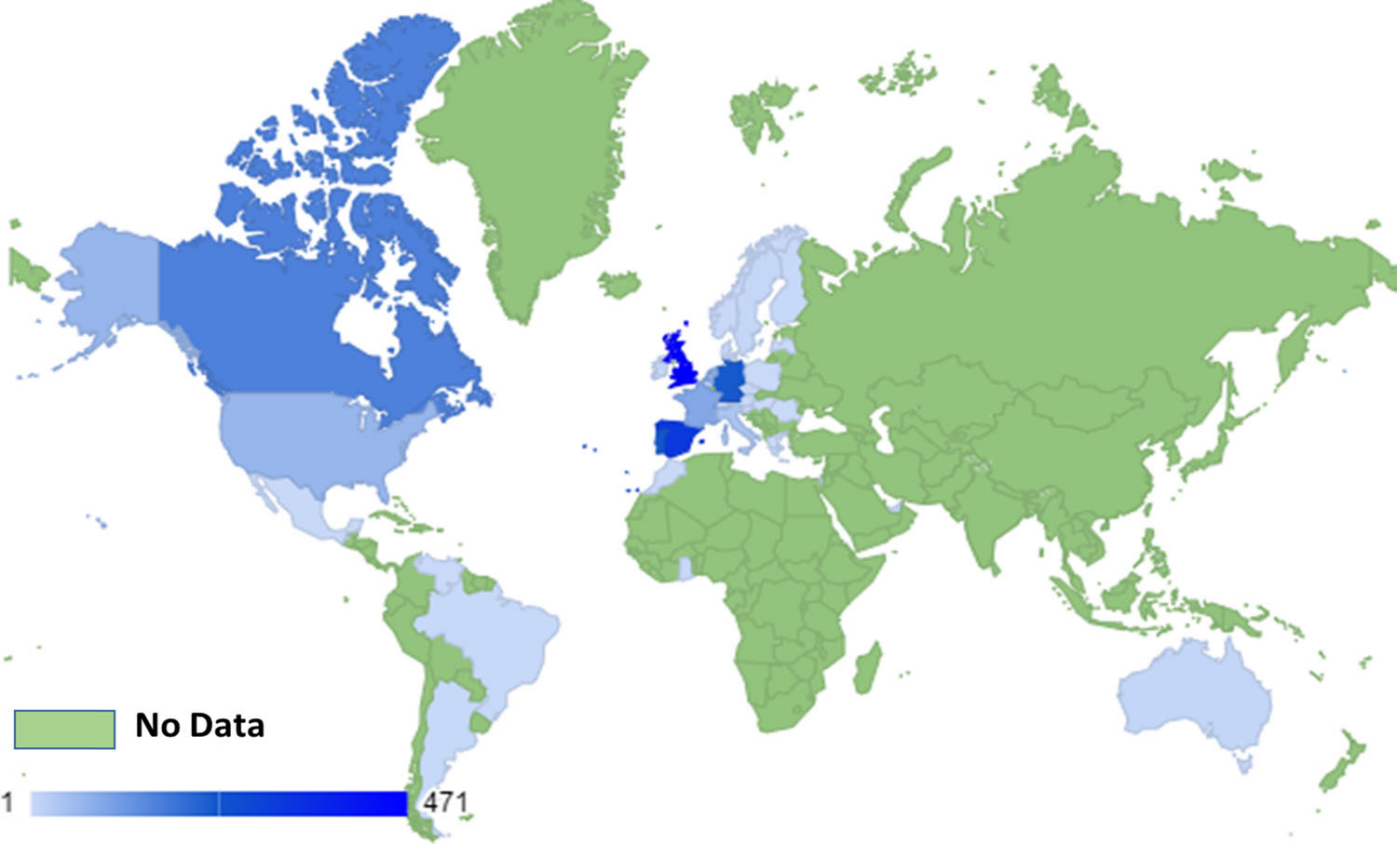
Global picture of Monkeypox confirmed case in nonepidemic countries till 14th of June, 2022.^4^

**Figure 2:**
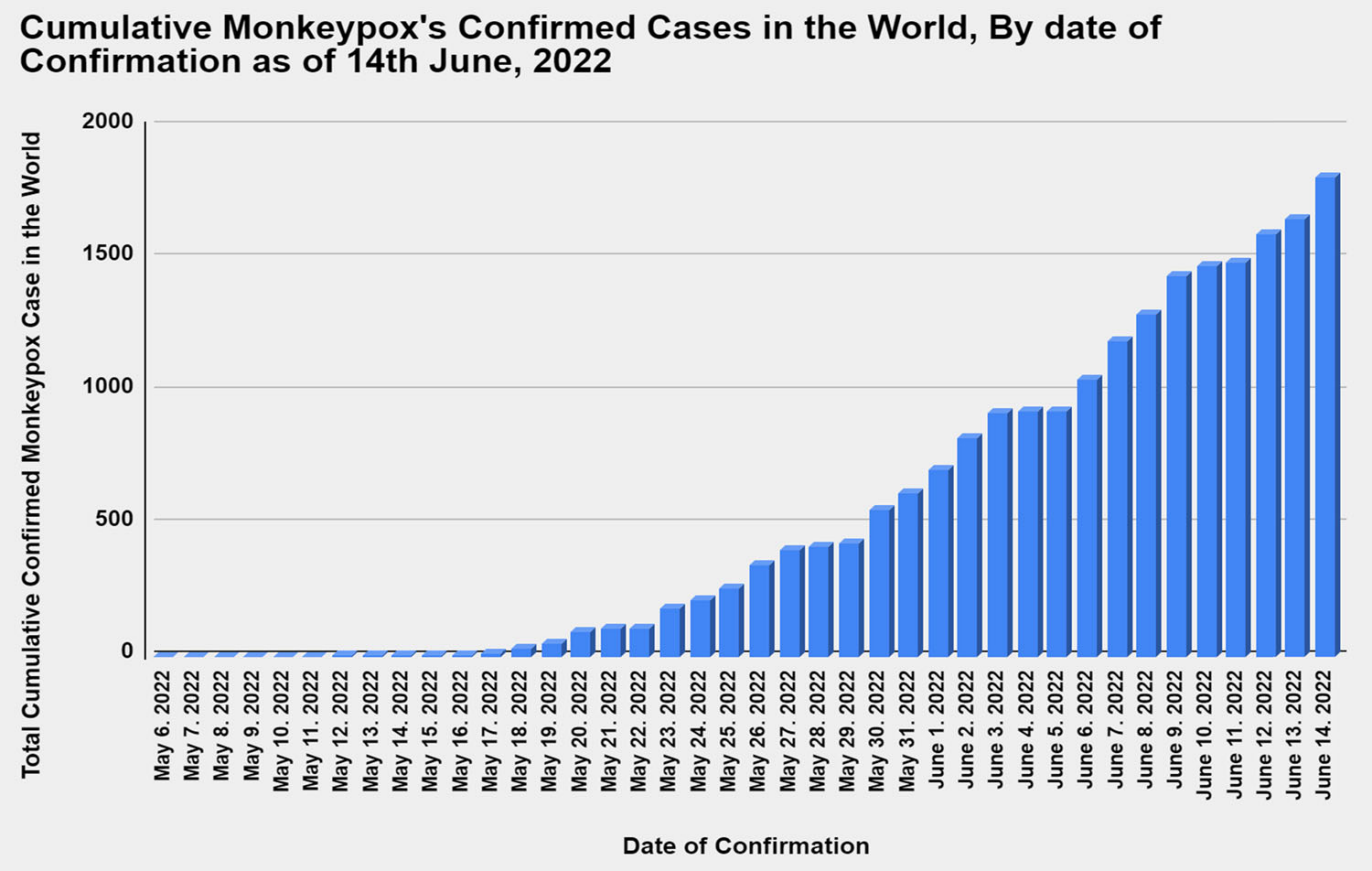
Daily incidence of reported confirmed monkeypox infections up until 14th June 2022.^4^

As of the 2nd of June 2022, 780 confirmed cases of Monkeypox have been reported in over 27 nonendemic countries.^1^ Figures 1 and 2 show the worldwide distribution of reported Monkeypox cases as of June 8th, 2021, and the daily increase in confirmed Monkeypox cases. Initially believed to be endemic only to Africa, this viral zoonotic illness has grabbed the attention of scientists globally to identify the reason for the rapid spread of the disease and the change in viral behavior.^2^, ^3^

The Human Monkeypox virus (MPXV) is a double‐stranded DNA virus belonging to the Orthopoxvirus genus of the Poxviridae family.^5^ Initially, detected in laboratory monkeys in 1958, Monkeypox was thought to transmit from wild animals like rodents or infected people through close contact with bodily fluids.^6^ It commonly presents with a 1–4‐day febrile prodrome with headache and fatigue followed by a centrifugal rash ranging from maculopapular, vesicular, or pustular. The spectrum of disease severity is varied, ranging from mild to even fatal with a mortality rate of 1%–10%.^7^ The emergence of the disease has been attributed to the waning of the herd immunity of variola or smallpox, which belongs to the same genus as MPXV.^5^ With the eradication of smallpox and subsequent discontinuation of the universal smallpox vaccination program in the 1970s, clusters of Monkeypox have been reported in Central and Western Africa, with the most recent outbreak in Nigeria in 2019.^8^ Although most affected individuals recover from Monkeypox without complications and treatment, there have been reports of a few fatal cases.

The recent outbreak of Monkeypox in non‐endemic regions of the world is of great concern. Monkeypox detection in individuals with no contact history or travel to endemic areas may suggest an asymptomatic disease state.^3^ Furthermore, increasing reported cases of transmission through sexual activity in men who have sex with men (MSM) is also concerning as MPXV was not known to be sexually transmitted.^9^ With growing evidence of globally confirmed cases we conducted this very first systematic review to understand better the current epidemiology, pathophysiology, clinical presentation and outcomes of Monkeypox.

## 2 METHODS

This systematic review was conducted and reported in conformity with the Cochrane and PRISMA (Preferred reporting items for systematic review and Meta‐analysis) 2020 guidelines as described previously.^10^, ^11^, ^12^

### 2.1 Search strategy

We conducted a systematic literature search in PubMed, Embase, Google Scholar and Scopus using predefined MESH terms by using “AND” and “OR”. The following search terms were used: Monkeypox [MeSH] OR “Monkeypox virus” [MeSH] OR “POX” OR “Monkeypox” AND “Outbreak” AND “Outcomes.”

### 2.2 Eligibility criteria

Studies were included if they fulfilled the following criteria: patients (of any age) who developed a rash and symptoms with a confirmed diagnosis of Monkeypox via swab or blood test, case reports, case series, prospective or retrospective studies. Studies which involved animal testing, review articles, modeling studies without patients’ data, and studies with smallpox as a diagnosis among the patient population were excluded.

### 2.3 Study selection

We queried databases from December 2019 till 14th June 2022 without language restriction. The studies were carefully screened and exported to the Endnote 2020 library (**X9**). Two reviewers (DM and SV) reviewed the titles and abstract. Discrepancies regarding inclusion of studies were arbitrated by the senior author (VJ). The same reviewers also performed the full‐text screening independently to decide which articles fulfilled the inclusion criteria. The senior author arbitrated discrepancies regarding the inclusion of studies.

### 2.4 Data extraction and statistical analysis

The following data were extracted from the studies: demographic data (age and gender), study design, publication year, study location, patient comorbidities, symptoms, length of hospitalization, duration of management, treatment used, and patient outcomes. Two authors (DM, SM) assembled all available information in a shared Excel® 2019 spreadsheet. For missing, incorrect, or unreported data, the corresponding authors of the respective papers were contacted via email for clarification. Supplementary material related to the main article was also investigated in such cases.

Finally, descriptive statistics were used to summarize the data in this paper. The mean and standard deviation were adopted to describe continuous variables, whereas frequencies and percentages were used for dichotomous data. All statistical analyses were conducted using the software R version 4.1.2 (available at https://cran.r-project.org/)

## 3 RESULTS

### 3.1 Study selection

Our initial search identified 438 articles. After removing 195 duplicates, 180 studies were excluded after a review of the title and abstract. 63 studies were sought for retrieval but 35 were not retrieved. A total of 28 studies were reviewed in full‐text form; however, 18 studies were further excluded based on inclusion criteria. A total of 10 studies were included for the review of which 4 were case reports,^13^, ^14^, ^15^, ^16^ 3 were case series,^17^, ^18^, ^19^ and 3 studies were observational^7^, ^20^, ^21^ (Figure S1).

### 3.2 Baseline characteristic of included patients

Ten studies were selected for the final review with a total of 1078 patients were identified to have symptoms suggestive of Monkeypox. A slight male predominance was found with (*n* = 583, 54.23%) male patients, and (*n* = 489, 45.48%) female patients. Mean age (±*SD*) of patients was 20.66 ± 16.45 years. Majority of patients (*n* = 1061, 98.4%) were from the Democratic Republic of Congo (DRC), (*n* = 10, 0.92% patients were from the United Kingdom), (*n* = 2, 0.18%) from Central Nigeria and West Africa, and (*n* = 1, 0.09) from United States of American, Australia, and Singapore (Tables 1, 2).

**Table 1:** Baseline patients characteristic of the included studies

Author	Study design	Year of publication	Country	Sample size	Confirmed cases	Suspected cases	Age, years	Male (M): female (F), *n*	Monkeypox viral DNA test
Adler et al.^7^	Retrospective observational	2022	United Kingdom	7	7	0	5 patients: 30–40 years; 1 patient: 40–50 years; 1 patient: less than 2 years	M: 4 F: 3	PCR
Eseigbe et al.^17^	Case series	2021	North Central Nigeria	2	2	0	20 years, 20 years	2M	PCR
Reynolds et al.^18^	Case series	2019	Sierra Leone, West Africa	2	2	0	11 months; 35 years	2M	PCR
Eltvedt et al.^13^	Case report	2020	Democratic Republic of the Congo	1	0	1	4 years	M	NR
Whitehouse et al.^20^	Surveillance data	2021	Democratic Republic of the Congo	1057	1057	0	14 years	M: 568, F: 486, NR: 3	PCR
Ngbolua et al.^21^	Cross sectional study	2020	Democratic Republic of the Congo	3	3	0	13, 7.7 years	3M	NR
Hobson et al.^19^	Case series	2021	United Kingdom	3	3	0	NR, NR, 18 months	NR	PCR
Yong et al.^15^	Case report	2020	Singapore	1	1	0	38 years	M	PCR
Costello et al.^14^	Case report	2022	United States	1	1	0	28 years	M	PCR
Hammerschlag et al.^16^	Case report	2022	Australia	1	1	0	30 years	M	PCR

**Table 2:** Summary table of all Monkeypox cases highlighting demographic, symptoms, management, and outcomes

Variables	*n*/*N* (%)
*N*	1078
Age (mean, *SD*)	20.66 ± 16.45 years
Male, *n* (%)	583 (54%)
Female, *n* (%)	489 (46%)
NR	6
Country	
United Kingdom	2/10 (20%)
Nigeria	1/10 (10%)
Sierra Leone	1/10 (10%)
Democratic Republic of the Congo	3/10 (30%)
Singapore	1/10 (10%)
United States	1/10 (10%)
Australia	1/10 (10%)
Monkeypox viral DNA test	RT‐PCR: 1074/1075 (99.9%)
Symptoms	
Fever	1037/1075 (96%)
Headache	1015/1068 (95%)
Lymphadenopathy	905/1070 (85%) (Cervical lymphadenopathy is most common)
Upper respiratory symptoms	1026/1060 (97%)
Rash	1078/1078 (100%)
Oral ulcers	1018/1057 (96%)
Vomiting	1011/1059 (95%)
Conjunctivitis	1017/1058 (96%)
Management	
Antivirals	7/11 (63%)
Antibiotics	4/10 (40%)
Length of hospitalization (mean)	13.3 ± 6.37 days
Duration of management (mean)	5 days
Outcomes	Recovery: 19/20 (95%), Death: 1/20 (5%)

### 3.3 Diagnostic test, symptoms, and clinical findings

A total of 1075 patients, 1074 (99.9%) patients tested positive for Monkeypox virus through RT‐PCR method, while 1 (0.09) patient was suspected. Symptoms were reported in all 10 studies. The major and most significant symptoms were rash 1078/1078 (100%), fever 1037/1075 (96%). Other important symptoms were upper respiratory symptoms 1026/1060 (97%), headache 1015/1068 (95%), vomiting 1011/1059 (95%), oral ulcers 1018/1057 (96%), conjunctivitis 1017/1058 (96%) and lymphadenopathy 905/1070 (85%). Most common lymphadenopathy is cervical lymphadenopathy. The appearance and distribution of rash was variably described in among the included studies with vesicular lesions being the most common presentation. The distribution was most often found to be generalized with an average of 158 lesions distributed over face and trunk also involving the limbs, palms, soles, and genitalia. Table 2 summarizes the symptoms experienced by the patients and the management in Monkeypox.

### 3.4 Management and patient outcomes

Four studies reported on the management of the patient. The information was available for 11 patients mainly. 4/10 (40%) patients received antibiotics in some form which included intravenous (IV) amoxicillin‐clavulanic acid, ceftriaxone, erythromycin ampicillin‐sulbactam, and antibacterial eye drops. 7/11 (63%) patients received antivirals (brincidofovir, tecovirimat, cidofovir, and acyclovir) and 9/12 (75%) patients underwent supportive treatment with isolation, IV fluids, analgesia, and oxygen therapy. Average duration of treatment was 5 days and mean hospitalization duration was 13.3 ± 6.37 days.

Patient outcomes were reported in 8 studies and for 20 patients.19/20 patients (95%) recovered fully from monkeypox, and 1/20 patient (5%) succumbed to the infection resulting in death (Table 2 and Table S1)

## 4 DISCUSSION

MPXV was first identified and isolated in 1958 as an outbreak of a pox‐like disease in monkeys in a research facility in Copenhagen, Denmark.^22^ It was first identified in humans in 1970 in a 9‐month‐old boy in the DRC who presented with a smallpox‐like disease.^23^ Since then, human cases have been increasingly reported in parts of central and western Africa, with numbers exceeding several thousand.^24^ Sporadic clusters have also been reported in regions outside of Africa. In 2003, infected domesticated prairie dogs in the Midwestern United States resulted in 53 human cases of Monkeypox.^25^ This cluster was identified as the first Monkeypox outbreak outside Africa and was caused by co‐inhabiting imported Gambian giant rats from Ghana.^25^ A few cases have also been sporadically reported in Israel, Singapore, United Kingdom, United States, and Canada from 2018 to 2021, all traced to preceding travel history to Nigeria.^26^ The exact incidence or prevalence of Monkeypox is unknown due to shortcomings in disease reporting and diagnostic confirmation in rural endemic areas of Africa. The identification of Monkeypox in several non‐African countries in May 2022 has propelled extensive research efforts to further understand disease epidemiology and pathophysiology.^24^

### 4.1 Evolving epidemiology of Monkeypox

Once thought to exclusively affect the pediatric population, this review revealed that Monkeypox is now also seen in adults with mean age of patient 20.66 ± 16.45 years with slight male predominance (54.23%). Similarly, a systematic review by Bunge et al.^6^ also noted a similar shift in median age of population affected by MPXV from 4 to 5 years old in the 1970s and 1980s to 10 and 21 years old in the 2000s and 2010s. Over the last five decades, the number of confirmed, probable, and/or potential monkeypox cases has increased by more than tenfold.^6^ This is in accordance with the findings of Hoff et al.,^27^ who concluded that the increase in monkeypox cases in the DRC from 2001 to 2013 was most likely due to significant disease increases rather than increased surveillance, given the monkeypox surveillance system was stable by 2008.

**Figure 3:**
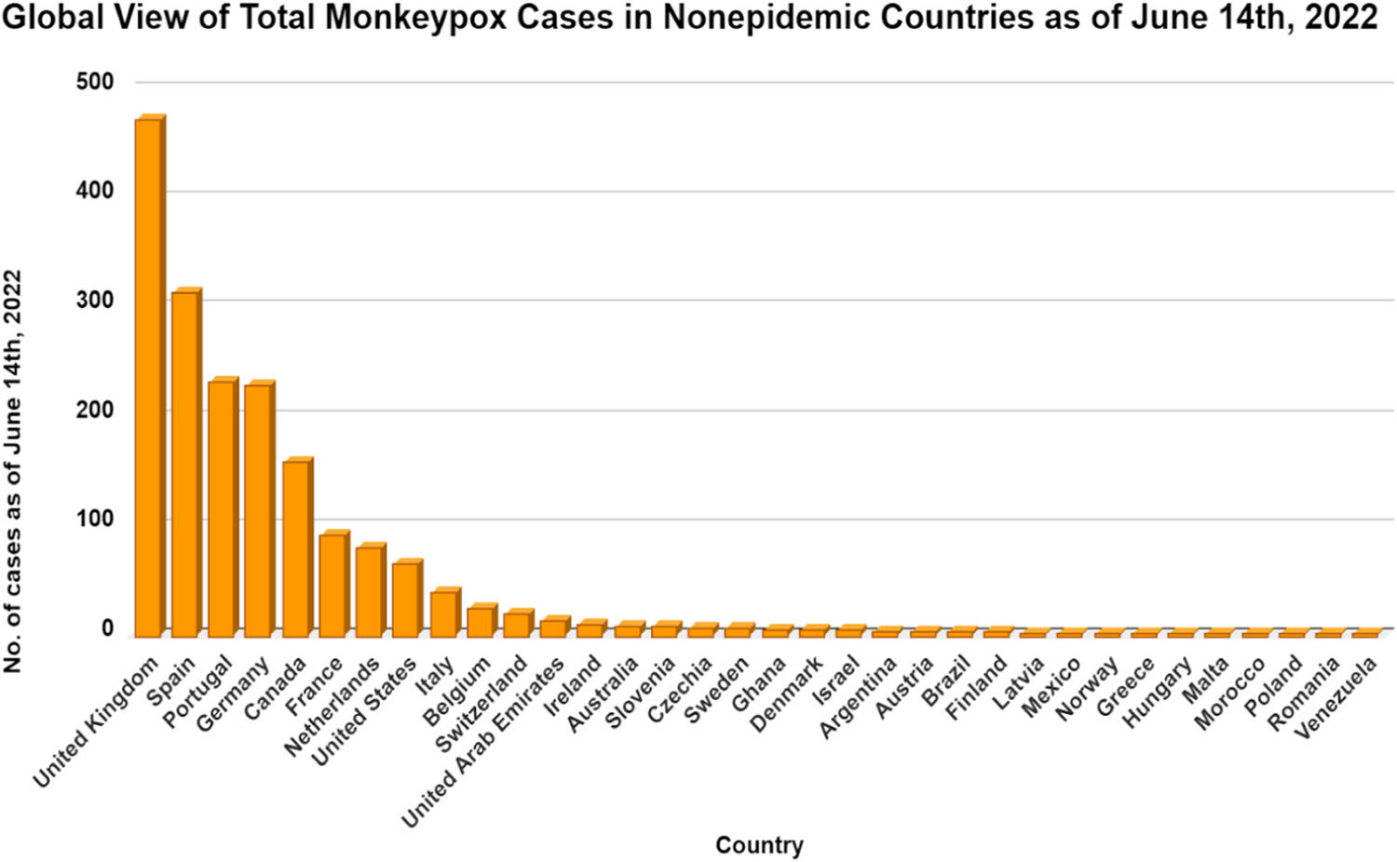
Distribution of the incidence Monkeypox cases globally by country^4^

MPXV is classified into two strains: the Congo Basin and the West Africa also called the Central Africa and West Africa clades based upon their geographical segregation.^28^ Congo basin clad was found predominantly in central African countries namely DRC,^5^ Central African Republic,^29^, ^30^ and South Sudan^31^ whereas the cases from United States,^32^ United Kingdom,^33^ Israel,^34^ Singapore,^15^ and the Nigerian epidemic^35^ are caused by West African clade. It is believed that West African clades are less virulent and have low transmissibility to humans when compared with Congo basin clades.^28^, ^36^ The Congo Basin clade virus has been linked to up to 10% mortality in monkeypox cases, whereas the West African clade is associated with fatal results in less than 1% of cases.^37^ 82.55% of total monkeypox cases reported from the DRC, previously thought to be a geographically limited zoonotic disease, have now spread in 27 other nonendemic countries across the^1^ globe. Figure 3 shows the nonendemic countries were Monkeypox was reported as of June 8th, 2021.

Monkeypox is a zoonotic disease with no known natural reservoir, however, certain rodents (including rope squirrels, tree squirrels, Gambian pouched rats, dormice) and nonhuman primates are known to be naturally susceptible to MPXV.^38^, ^39^ At present it is unclear if animals outside of the African continent can maintain a zoonotic cycle of MPXV.^38^ However, it is thought that reintroduction of MPXV into animals on a regular basis is essential to keep the disease alive in the human population.^40^ Despite the role of reservoir species in transmission, research shows that environmental variables such as warm temperature and humid environment have a positive influence on MPXV survival outside a host.^31^, ^41^, ^42^ The sudden uproar of monkeypox cases in numerous nonendemic nations globally during the same period without a known animal reservoir or an established travel history to endemic regions hints towards an undiscovered transmission for an unknown period of time, followed by recent amplifier occurrences.

### 4.2 Pathophysiology of Monkeypox

MPXV pathophysiology is like that of smallpox. After initial viral entry through the skin or respiratory system, the virus spreads to regional lymph nodes and begins to replicate. The initial signs of viremia appear 3–4 days after infection and the virus further spreads within the lymphatic system. The emergence of fever and clinical symptoms of sickness correlates with the onset of secondary viremia, which occurs between days 8 and 12 following infection. At this stage, the virus has become localized in the oropharyngeal mucosa and small blood vessels of the dermis resulting in the appearance of a rash and myriad of other clinical symptoms.^43^ Appearance of a rash in a patient generally indicates the start of the infectious period; however, the Centers for Disease Control and Prevention (CDC) states that a patient can be contagious throughout the prodromal stage.^44^ The incubation period is about 12 days long,^45^ but it can last up to 21 days.^44^

### 4.3 Clinical features and differential diagnosis of Monkeypox

Overwhelmingly, the clinical features of monkeypox are like those of smallpox. More than 90% patients of monkeypox clinically present with a fever, rash, headache, and upper respiratory symptoms and more than 80% patients have lymphadenopathy, oral ulcers, conjunctivitis, vomiting and diarrhea as noted by this review. Initial symptoms of both smallpox and monkeypox include febrile illness along with the appearance of vesiculopapular rash which is umbilicated and evolves at similar stages of development throughout the illness.^46^ Smallpox lesions are deep seated in contrast to superficial lesions of monkeypox.^46^ In majority of monkeypox cases, the rash usually starts on the face and spreads out in a centrifugal pattern across the body including oral mucosa, trunk limbs, palms, soles and genitalia.^20^ The rash begins with macules, which then develop into papules, vesicles, pustules, and finally crusts.^45^, ^47^, ^48^

Another close differential of monkeypox is chickenpox. Monkeypox rash is differentiated from chickenpox rash by virtue of its slower maturation rate. Chickenpox lesions are more superficial, smaller, and centrally oriented, as opposed to the centrifugal distribution of Monkeypox, and they classically evolve in “crops” over 3–5 days, compared to the typical 12 days for Monkeypox. Presence of lymphadenopathy can help clinicians to differentiate monkeypox from smallpox and chickenpox as well.^45^ Numbers of lesions in a monkeypox patient are highly variable, ranging from few to thousands with an average of 158 lesions. Monkeypox generally presents with diffuse multiple skin rash, however, a patient who had direct contact with an infected prairie dog, presented with prodromal symptoms followed by lymphadenopathy and later tested positive for MPXV in serologic testing had only one skin lesion.^49^ This makes relying only on clinical presentation of patients for the diagnosis of monkeypox unreliable. For definitive diagnosis of MPXV positive polymerase chain reaction or next generation sequencing in a clinical specimen or isolation of *Monkeypox virus* in culture from a clinical specimen is required.^50^ This increases challenges for physicians in low‐income hospital setups of West and central Africa where monkeypox is endemic. A frequent dilemma physicians face is differentiating Monkeypox from chickenpox in patients with atypical presentation. In low‐income countries, serum anti‐orthopoxvirus IgM antibody tests can be sufficient for diagnosis if the patient has not been exposed to another of the same genus.^50^

Common complications of monkeypox include bronchopneumonia, encephalitis, septicemia, corneal scarring, blindness, skin scarring and severe dehydration.^5^ Young children, unvaccinated and immunocompromised individuals are at higher risk of poorer outcomes. The case fatality rate varies widely from 0% to 11% depending on the outbreak, but according to the present knowledge the CDC estimates it to be around 10% in Africa.^44^

### 4.4 Spread of Monkeypox in COVID era

Transmission of MPXV is via contact with infected animal, person or materials contaminated with virus.^45^ It can be transmitted from animals to humans by infected animal bite or scratch, handling wild game, or using infected animal products.^45^ It is now well known that MPXV spreads via large respiratory droplets as well.^45^, ^51^ It is noteworthy here that MPXV spread between humans is similar to that of coronavirus COVID‐19 hence making it a global health concern. Monkeypox has epidemic potential, according to mathematical modeling of human‐to‐human transmission, with R0 > 1^52^ thus raising concerns how infected visitors might serve as index cases for local epidemics. An individual can become infected with MPXV related to sexual or nonsexual contact. Nonsexual contact includes direct contact with the rash, sores, or scabs of infected individuals, contact with objects or surfaces contaminated by infected individuals, or through respiratory droplets or oral fluids of infected individuals.^45^, ^53^ Spread of MPXV through sexual contact has received considerable attention after evidence from recent studies points towards a higher prevalence of MPXV infection in MSM. A study from the United Kingdom reported 54 MPX cases and noted that all the cases were MSM.^54^ Another study from Italy documented four cases of MPX in MSM who reported having unprotected sexual intercourse.^55^ A recent review of 121 cases of MPX highlighted that sexual exposure could be attributed to over 91% of the cases, which was supported by the findings of another study that included 528 individuals infected by MPX, which revealed that 98% of the confirmed MPX cases were either gay or bisexual.^56^, ^57^ Sexual transmission of MPX is an alarming threat to the spread of MPXV, however, more extensive studies confirming the presence of MPX in seminal samples through cell cultures and viral isolation are necessary to ascertain this mode of transmission.

### 4.5 Recommendations

It is past time for us to begin taking steps to prevent and prepare for epidemics, particularly for viruses that have been identified as substantial human dangers, such as MPXV. Even though it was identified in 1958 and first documented in humans in 1970, no gold standard treatment or vaccine exists. Current treatment guidelines focus on patient isolation, monitoring and treatment of complications and symptomatic treatment. Development of appropriate and effective treatment therapies, as well as active case surveillance, is critical to prevent another global pandemic. Measures for frequent surveillance among suspected animal reservoirs should be implemented to prevent repeated outbreaks. Repeated epidemics may result in a more lethal virus because of genetic recombination hence swift actions by the public health authorities is required. For the last 2 years, Healthcare professionals and the general public are successfully daily practicing airborne precautions and hand hygiene to curb spread of COVID‐19. Similar public education strategies can be used to control the monkeypox epidemic. COVID‐19 pandemic has highlighted the crucial role the research community plays in handling a public health emergency. As seen by the findings in this review, the information about monkeypox disease is still inadequate and fragmented, thus requiring urgent attention of research groups to successfully prevent another pandemic by a deadly virus.

## 5 CONCLUSION

The recent outbreak of Monkey in over 27 nonendemic countries is of great concern globally, especially following the COVID‐19 pandemic. The results of this review indicate that recent cases of Monkeypox have been occurring in adults and not only in children. The symptoms were as expected with a predominance of rash, fever, headache, upper respiratory symptoms and lymphadenopathy. Transmission of the disease can be either by contact with infectious sores, scabs, or body fluids or respiratory droplets, similarly to COVID‐19, therefore, similar preventative measures can prevent the disease from becoming epidemic/pandemic. Additionally, recent cases in MSM could suggest a sexual transmission of the virus which requires further indepth research to rule out. The findings of this review highlight that the information about monkeypox disease (clinical features and transmission) is still inadequate, thus necessitating further research to successfully prevent the spread of the virus.

## AUTHOR CONTRIBUTIONS

*Conceptualization*: Vikash Jaiswal. *Methodology*: Vikash Jaiswal. *Study Screening and data extraction*: Mittal Savaliya. *Formal analysis and investigation*: Vikash Jaiswal, Dattatreya Mukherjee, Nitya Batra. *Images and illustrations work*: Mittal Savaliya, Vikash Jaiswal. *Writing—original draft preparation*: Vikash Jaiswal, Priyanshu Nain, Dattatreya Mukherjee, Amey Joshi, Mittal Savaliya, Angela Ishak, Nitya Batra, Nishan Babu Pokhrel. *Writing—review and editing*: Vikash Jaiswal, Angela Ishak, Dattatreya Mukherjee, Nishan.

## CONFLICTS OF INTEREST

The authors declare no conflicts of interest.

## Supporting information

Supporting information.
