# Corrigendum

**DOI:** 10.1002/iid3.774

**Published:** 2023-01-23

In Jaiswaled et al.,^1^ authors' discovered that the percentage values were incorrectly calculated. The values have been corrected to update percentage values in different section of the paper.

The authors apologize for this error.

In **Results** the symptoms recorded read “The major symptoms were rash (100%), fever (96%), and other important symptoms were upper respiratory symptoms (97%), headache (95%), vomiting (95%), oral ulcers (96%), conjunctivitis (96%), and lymphadenopathy (85%).”

They should have read: “The major symptoms were rash (100%), fever (99%), and other important symptoms were upper respiratory symptoms (55%), lymphadenopathy (85%), headache (78%), vomiting (25%), oral ulcers (56%), and conjunctivitis (21%).”

In **Paragraph 3.3** the reported symptoms read “The major and most significant symptoms were rash 1078/1078 (100%), fever 1037/1075 (96%). Other important symptoms were upper respiratory symptoms 1026/1060 (97%), headache 1015/1068 (95%), vomiting 1011/1059 (95%), oral ulcers 1018/1057 (96%), conjunctivitis 1017/1058 (96%), and lymphadenopathy 905/1070 (85%).”

They should have read: “The major and most significant symptoms were rash 1078/1078 (100%), fever 1037/1047 (99%). Other important symptoms were upper respiratory symptoms 563/1027 (55%), headache 797/1021 (78%), vomiting 251/1012 (25%), oral ulcers 570/1018 (56%), conjunctivitis 211/1017 (21%), and lymphadenopathy 886/1047 (85%).”

In **Paragraph 4.3** the clinical features read “Overwhelmingly, the clinical features of monkeypox are like those of smallpox. More than 90% patients of monkeypox clinically present with a fever, rash, headache, and upper respiratory symptoms and more than 80% patients have lymphadenopathy, oral ulcers, conjunctivitis, vomiting, and diarrhea as noted by this review.”

They should have read “Overwhelmingly, the clinical features of monkeypox are like those of smallpox. Patients with monkeypox clinically present with a fever, rash, headache, lymphadenopathy, and upper respiratory symptoms and other less common symptoms which include oral ulcers, conjunctivitis, vomiting, and diarrhea as noted by this review.”

In Supporting Information: Table 1, affected values have been corrected and uploaded online.

Table 2 has been updated with the recalculated percentage points, shown in red.

**Table 2:** Summary table of all monkeypox cases highlighting demographic, symptoms, management, and outcomes.

Variables	*n*/*N* (%)
*N*	1078
Age (mean, SD)	20.66 ± 16.45 years
Male, *n* (%)	583 [54%]
Female, *n* (%)	489 [46%]
NR	6
Country	
UK	2/10 [20%]
Nigeria	1/10 [10%]
Sierra Leone	1/10 [10%]
DR Congo	3/10 [30%]
Singapore	1/10 [10%]
USA	1/10 [10%]
Australia	1/10 [10%]
Monkeypox viral DNA test	RT PCR: 1074/1075 [99.9%]
Symptoms	
Fever	1037/1047 [99%]
Headache	797/1021 [78%]
Lymphadenopathy	886/1047 [85%] [cervical lymphadenopathy is most common]
Upper respiratory symptoms	563/1027 [55%]
Rash	1078/1078 [100%]
Oral ulcers	570/1018 [56%]
Vomiting	251/1012 [25%]
Conjunctivitis	211/1017 [21%]
Management	
Antivirals	7/11 [63%]
Antibiotics	4/10[40%]
Length of hospitalization (mean)	13.3 ± 6.37 days
Duration of management (mean)	5 days
Outcomes	Recovery‐ 19/20 [95%] Death: 1/20 [5%]
