# Supplementary material for: Symptomatology, prognosis, and clinical findings of Monkeypox infected patients during COVID‐19 era: A systematic‐review
Source: Immun Inflamm Dis. 2022 Oct 11;10(11):e722. doi: 10.1002/iid3.722 (PMC9552975; doi:10.1002/iid3.722)
Supplement: Supplementary file 1 — Supporting information. [file IID3-10-e722-s001.docx]

**Supplementary File**

**Symptomatology, prognosis, and Clinical findings of Monkeypox infected patients during COVID-19 era: A Systematic-review**

**Authors:** Vikash Jaiswal MD, Priyanshu Nain MBBS, Dattatreya Mukherjee MBBS, Amey Joshi MBBS, Mittal Savaliya MBBS, Angela Ishak MD, Nitya Batra MD, Nishan Babu Pokhrel MD.

**Supplementary Figure 1:** PRISMA Flow of the search strategy for systematic review


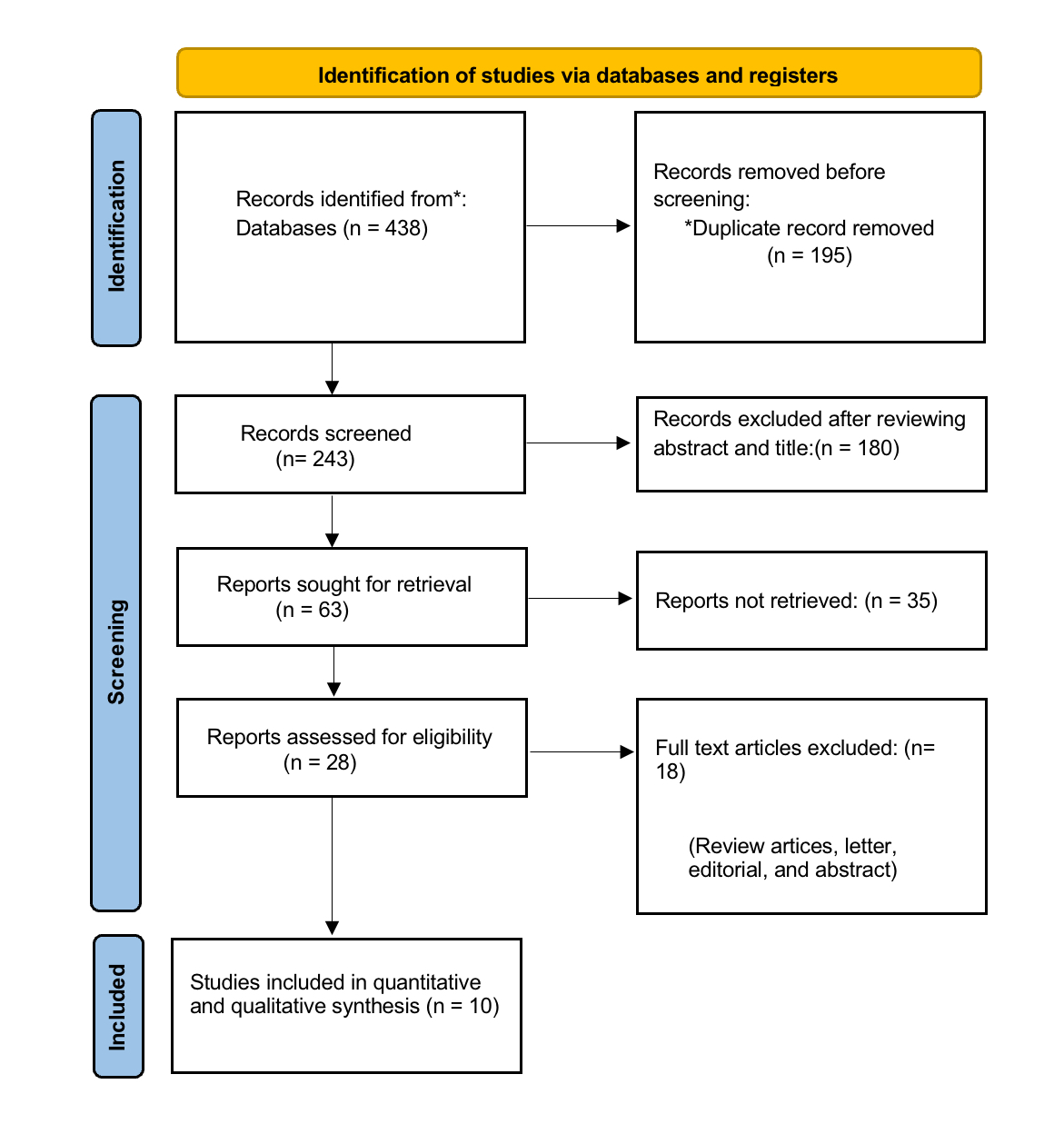


**Supplementary Table 1:**  Symptoms and Management in Monkeypox

| **Author** | **Prodrome** | **Fever** | **Myalgia** | **Headache** | **Rash** | **Localisation of Rashes** | **Lesion Count** | **Lymphadenopathy** | **Management** | **Outcome** | **Post Outcome Complication** |
| --- | --- | --- | --- | --- | --- | --- | --- | --- | --- | --- | --- |
| Adler et al  [6] | Fever:3  Headache:1  Groin swelling:1  Night sweats:1  Coryzal illness:1 | 3 | NR | 1 | Yes: 7 | Face:7  ,Trunk:7, Arms:7,Legs:7, Soles:7, Palms:7, Glans:7, Labia:7 | 3 Pts- ≥ 100 ;  4 pts ≤ 50 | 5 patients | Antivirals:7 complications management:7 | Recovered: 7 | NR |
| Eseigbe et al  [16] | Fever: 2 Headache: 2  Pain on Swallowing:2  Pain on Micturition: 1 | 2 | NR | 2 | Yes: 2 | Generalised Rash: 2 | NR | NR | Antibiotics:2 Antihistamine:2,  NSAID:2, Multivitamins:2 | Recovered: 2 | NR |
| Reynolds et al  [17] | Fever: 2  Rash: 2  Sweats: 1 Chills:1, Vomiting:1, Loss of appetite, Cough:1, Pruritus:1, Generalized body pain:1 Dysphagia:1  Malaise:1 | 2 | 1 | NR | Yes:2 | Face:1, Mouth:1, Oral Mucosa:1, Trunk:1, Back:1, Palms:1, Genital area:1, | NR | Enlarged cervical lymph nodes:1 | NR | Recovered:2 | NR |
| Eltvedt et al  [12] | Fever  Rhinitis, Conjunctivitis  Cough | 1 | NR | NR | Yes:1 | Truncal area:1 face:1, Palms:1,  Foot:1, Soles:1, Mucous membranes:1 | NR | Cervical lymphadenitis:1 | IV Amoxicillin-  Clavulanic acid:1, Retinol tablets:1, Antibiotic eye drops:1, Paracetamol:1, Diluted plumpy nut:1, ,IV. maintenance fluids:1. | Death | NR |
| Whitehouse et al  [19] | Fever: 1023  Vomiting: 1010  Cough: 1024  Chills: 1027  Dysphagia: 1032  Buccal ulcer: 1018 Headaches: 1011 Itching: 1012, Myalgia: 1003  Fatigue: 1029, Conjunctivitis 1016  Photophobia: 999  Bedridden status: 1021 | 1023 | 1003 | 1011 | Yes: 1057 | Face:  1036, Trunk:  1028, Arms: 1026, Palms:  1009, Legs: 786, Soles 885, Genitals: 300 | <25 Lesion in 48 pt; 26 -100 in 462 pts; 101-249 in 392 pts, >250 Lesion in 141 pts | 84.7% pt | NR | NR | NR |
| Ngbolua et al [20] | Fever: 3  Skin eruption: 2 Pruritus:2  Abdominal pain: 1 | 3 | NR | NR | Yes:3 | Whole body:3 | NR | NR | NR | Recovered: 3 | NR |
| Hobson et al [18] | NR | NR | NR | NR | Yes: 3 | NR | NR | NR | NR | Recovered: 3 | NR |
| Yong et al  [14] | Fever:1  Chills:1  Myalgia:1 | 1 | 1 | NR | Yes: 1 | Face:1, Trunk:1, Palms:1 Soles:1, Penile Shaft:1, Glans Penis:1, Limbs:1 | NR | Cervical and Inguinal Lymph nodes:l | NR | Recovered: 1 | NR |
| Costello et al [13] | Fever:1  Chills:1  Headache:1 | 1 | NR | 1 | Yes | Face:1, Arms:1, Trunk:1, Inner thigh:1 | NR | Cervical:Lymph nodes 1 | NR | NR | NR |
| Hammerschlag et al  [15] | 1st Genital Rash- painless pustules on the penis which became painful and pruritic, Fever, malaise, rash on face and limb | 1 | 0 | 0 | Yes | Penis:1, Face:1 Limb:1 | NR | Present | Ceftriaxone:1  Oral Doxycycline:1,  Contact and airborne:1 precaution, Isolation in negative pressure room:1 | Recovered | NR |
